# Prediction Model for Critically Ill Patients with Acute Respiratory Distress Syndrome

**DOI:** 10.1371/journal.pone.0120641

**Published:** 2015-03-30

**Authors:** Zhongheng Zhang, Hongying Ni

**Affiliations:** Department of Critical Care Medicine, Jinhua Municipal Central Hospital, Jinhua Hospital of Zhejiang University, Zhejiang, P.R. China; Azienda Ospedaliero-Universitaria Careggi, ITALY

## Abstract

**Background and objectives:**

Acute respiratory distress syndrome (ARDS) is a major cause respiratory failure in intensive care unit (ICU). Early recognition of patients at high risk of death is of vital importance in managing them. The aim of the study was to establish a prediction model by using variables that were readily available in routine clinical practice.

**Methods:**

The study was a secondary analysis of data obtained from the NHLBI Biologic Specimen and Data Repository Information Coordinating Center. Patients were enrolled between August 2007 and July 2008 from 33 hospitals. Demographics and laboratory findings were extracted from dataset. Univariate analyses were performed to screen variables with p<0.3. Then these variables were subject to automatic stepwise forward selection with significance level of 0.1. Interaction terms and fractional polynomials were examined for variables in the main effect model. Multiple imputations and bootstraps procedures were used to obtain estimations of coefficients with better external validation. Overall model fit and logistic regression diagnostics were explored.

**Main result:**

A total of 282 ARDS patients were included for model development. The final model included eight variables without interaction terms and non-linear functions. Because the variable coefficients changed substantially after exclusion of most poorly fitted and influential subjects, we estimated the coefficient after exclusion of these outliers. The equation for the fitted model was: g(Χ)=0.06×age(in years)+2.23(if on vasopressor)+1.37×potassium (mmol/l)-0.007×platelet count (×10^9^)+0.03×heart rate (/min)-0.29×Hb(g/dl)-0.67×T(°C)+0.01×PaO_2+13, and the probability of death π(Χ)=e^g(Χ)^/(1+e^g(Χ)^).

**Conclusion:**

The study established a prediction model for ARDS patients requiring mechanical ventilation. The model was examined with rigorous methodology and can be used for risk stratification in ARDS patients.

## Introduction

Acute respiratory distress syndrome (ARDS) is a severe form of acute lung injury most commonly seen in intensive care unit (ICU) and it is associated with significant morbidity and mortality. The incidence of ARDS is estimated to be approximately 40–80 per 100,000 patient-years [1–4]. and the figure can vary with different definitions of ARDS. The mortality rate in patients with established ARDS is around 50–60% [4–6]. More recently, due to advances in the management of ARDS such as use of low tidal volume ventilation and extracorporeal membrane oxygenation, the mortality has shown a reduction to around 30% [7, 8]. However, There is limited supportive evidence that specific interventions can decrease mortality in ARDS, and the mortality of ARDS does not show significant reduction over time.[9–12] Therefore, ARDS remains to be a great challenge to clinicians.

The initial step in mortality reduction is to identify risk factors for poor clinical outcomes. This is an area being extensively studied. For instance, sepsis-induced ARDS has been found to be associated with increased risk of death as compared with other caused.[13] Patients with lower BAL levels of procollagen peptide showed lower mortality than those with higher levels.[14] However, most of these studies investigated risk factors in isolation. Because there are multiple factors working together to determine the final outcomes of ARDS patients, it is more clinically useful to develop a prediction model for risk stratification. Gajic O and colleagues[15] developed a well-calibrated model for mortality prediction for ARDS patients, which however required information on organ functions three days after intubation. In another study, risk tertiles model was developed for predicting mortality in ARDS. However, the study categorized continuous variables into tertiles, which is thought to be associated with information loss [16]. In the present study, we aimed to develop a prediction model for ARDS patients requiring mechanical ventilation. The principal in developing the model is a balance between parsimony and model fitting. Furthermore, the variables included in the model should be readily available in routine clinical practices.

## Methods

The performance of the secondary data analysis was approved by the ethics committee of Jinhua municipal central hospital and informed consent was waived. Patient records or information was anonymized and de-identified prior to analysis. The study was a secondary analysis of data obtained from the NHLBI Biologic Specimen and Data Repository Information Coordinating Center. The original randomized controlled trial was entitled “Randomized, Placebo-controlled Clinical Trial of an Aerosolized b2-Agonist for Treatment of Acute Lung Injury” (NCT 00434993) and has been published elsewhere.[17]

Patients were enrolled between August 2007 and July 2008 from 33 hospitals of the National heart, lung, and blood institute ARDS clinical trials network. Inclusion criteria were 1) patient had to be intubated and receiving mechanical ventilation; 2) bilateral infiltrates consistent with edema on chest X-ray, 3) had an PaO2/FiO2<300, 4) no clinical evidence of left atrial hypertension. The definition of ARDS in the study was made according to the American-European Consensus Conference (AECC) criteria [18]. In Berlin definition, the use of PEEP was considered and ARDS was categorized into mild, moderate and severe forms [19]. Patients were excluded if 1) they had coexisting chronic lung disease; 2) unable to obtain consent; 3) acute myocardial infarction; 4) chronic liver disease; 5) neuromuscular disease.

### Data extraction

Data were extracted from the dataset obtained from the NHLBI Biologic Specimen and Data Repository Information Coordinating Center after approval. The variable name was annotated in a file named “data dictionary”. The following variables were extracted: age, gender, body mass index (BMI), past history of cigarette smoking and alcohol consumption, types of ICU (medical ICU, surgical ICU and mixed ICU), admission type (unscheduled surgery, scheduled surgery and medical admission), causes of ARDS (sepsis, transfusion, aspiration, pneumonia, other lung conditions), admission source (operating room, emergency department, floor ward and others), comorbidities (chronic dialysis, leukemia, immunodeficiency, cirrhosis, diabetes, hypertension, myocardial infarction, heart failure, vascular disease, dementia, chronic pulmonary disease, arthritis, peptic ulcer), vasopressor use, hemoglobin, white blood cell, platelet count, creatinine, bilirubin, sodium, potassium, glucose, bicarbonate, phosphate, magnesium, total protein, albumin, minimal glucose, FiO2, PaO2, PaCO2, pH, lowest and highest temperature, lowest and highest systolic blood pressure, lowest and highest mean blood pressure, lowest and highest respiratory rate, urine output (24 hours), transfusion of RBC, FFP transfusion. All measurements, including laboratory and physiological findings were performed within the first 24 hours.

### Statistical analysis

Univariate logistic regression model was performed to screen factors associated with mortality in ARDS patients requiring mechanical ventilation. The dependent variable was a binary outcome with “1” indicated death and “0” indicated survival. Independent variables were categorized into continuous variables and indicator variables. Continuous variables such as age, laboratory measurements and urine output were reported their ORs for each one unit increase in the parameter. Indicator variables were reported their ORs for each category as referenced to the base status. For instance, patients exposed to cigarette smoking were compared to those without cigarette smoking, and OR was reported for the variable. Alcohol intake was reported as the frequency and we dichotomized them into patients with and without history of alcohol intake. Smokers were reported as non-smoker, former smoker and current smoker. We combined the latter two categories as smokers. There were eight categories for the variable of admission sources. Because too many categories might compromise the statistical power, we grouped patients from operating room and recovery room together.

Variables with p< 0.3 in univariate analysis are included for automatic stepwise selection of covariates. Furthermore, variables with prevalence<10% or with >15% missing observations were excluded from further analysis. In the study, phosphate and bilirubin both showed more than 30% missing observations and was excluded from multivariable analysis despite their significance in univariate analysis. Stepwise forward selection of variable with p<0.1 was performed to screen variables independently associated with mortality. The automatic selection would finally generate a main effect model. To make full use of data information and improve statistical power, we use multiple imputation technique other than the conventional complete case analysis as a sensitivity test to examine the robustness of our result.[20] Overfitting with optimism in coefficient estimate was another concern in building a prediction model.[21] Thus, we use the bootstrapping technique to adjust for coefficients estimated from conventional multivariable analysis.[22] Bootstrapping was a technique of resampling with replacement and here we repeated the sampling for 500 times. The bootstrapping sampling technique would shrink the estimated coefficient but provide better prediction to future samples.

Potential interactions among included covariates in the main effect model were tested by including them one by one. To simplify this process, we created a local macro called “covariate” to store all covariates in the main effect model and thereafter the process can be automated by using *foreach* syntax. Interaction terms with p<0.1 would be included in the model. Linearity of covariates on logit scale is a fundamental assumption in model fitting. Therefore, we employed multivariable fractional polynomials (MFP) to test whether other power terms were superior to the linear term.[23, 24] Firstly, the best fitting one-term and two-term models were modeled by choosing power transformations from the set <-2, -1, -0.5, 0, 0.5, 1, 2, 3>, where 0 denoted the log transformation. Next, the closed test procedure was performed in which the best fitting two-term model was compared with the linear model. If the two-term model was significantly better than the linear one (p<0.05), two-term model was then compared to the best fitting one-term model. Otherwise, linear model was adopted. The procedure continued until there was no statistical significance and the best fitting model was chosen.

Model fit would be assessed from two aspects: a summary measure and regression diagnostics. The Hosmer-Lemeshow tests would be employed in which grouping of covariate pattern was based on the estimated probability.[25] The Hosmer-Lemeshow goodness-of-fit statistic was obtained by calculating Pearson Chi-square statistic from the g×2 table of observed and estimated frequencies. The variable g refers to the number of groups. A statistical significance level p<0.05 indicates that the model is significantly different from the observed outcome. Furthermore, the discrimination of the fitted model was assessed graphically. Logistic regression diagnostics were calculated to see if the model fit over the entire set of covariate patterns.[26] Statistics including leverage, change in Pearson Chi-square (Δχ^2^), change in deviance (ΔD) and Cook’s distance (Δ$\hat{\beta }$) would be plotted against the estimated probability of death ($\hat{\pi }$).[27] The aim was to examine cases lied far away from the others. New model fitting would be performed by excluding these outliers. However, diagnostics statistics were used to identify influential subjects and the decision on exclusion should incorporate subject matter considerations.

All statistical analyses were performed by using the STATA 13.1 (StataCorp, College Station, Texas 77845 USA).

## Results

**Fig 1:**
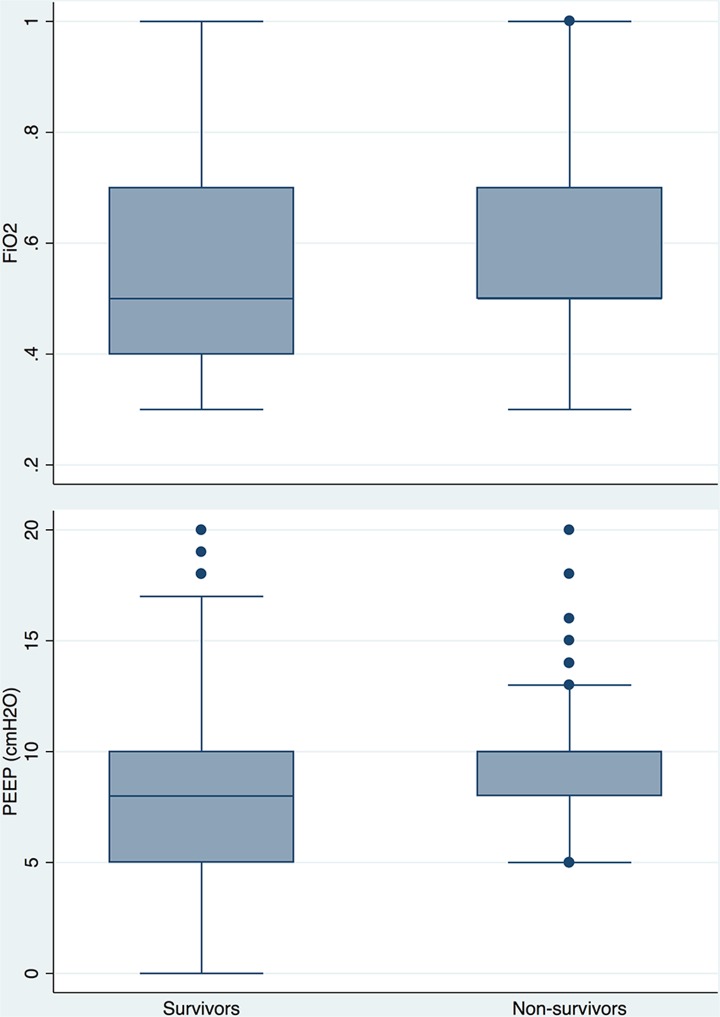
Graphical presentation of PEEP and FiO2 in survivors and non-survivors at the onset of ARDS. There was no significant difference on PEEP (9.1±3.7 vs.9.3±3.3 cmH2O; p = 0.71) and FiO2 (0.58±0.19 vs.0.57±0.17; p = 0.69) between survivors and non-survivors.

A total of 2688 patients were initially screened. Then 2406 patients were excluded, remaining 282 ARDS patients who required invasive mechanical ventilation. The most common reasons for exclusion were chronic lung disease (19.2%), unable to obtain consent (15.2%) and time window exceeded (14.7%). There were 61 non-survivors and 221 survivors, with an overall mortality rate of 21.63%. Univariate logistic regression analysis (Table 1) showed that age (OR: 1.04, 95% CI: 1.02–1.06), admission from floor ward (OR: 3.70, 95% CI: 1.02–13.46), leukemia (OR: 7.68, 95% CI: 1.37–43.01), vascular disease (OR: 11.38, 95% CI: 1.16–111.42), vasopressor use (OR: 5.08, 95% CI: 2.60–9.92), platelet count (OR: 0.996, 95% CI: 0.993–0.999), potassium (OR: 1.98, 95% CI: 1.27–3.08), bicarbonate (OR: 0.93, 95% CI: 0.88–0.98), phosphate (OR: 1.40, 95% CI: 1.09–1.81), pH (OR: 0.028, 95% CI: 0.002–0.41), lowest temperature (OR: 0.70, 95% CI: 0.53–0.93), highest temperature (OR: 0.63, 95% CI: 0.45–0.88), lowest systolic pressure (OR: 0.98, 95% CI: 0.96–0.99), urine output (OR: 0.9997, 95% CI: 0.9995–0.9999). Five variables contained missing values: bilirubin (49), phosphate (129), PaO2 (9), pH (9), mean blood pressure (2), urine output (2). Because the variables bilirubin and phosphate had too many missing values (>15%), they were excluded from analysis. Furthermore, the comorbidity variables including leukemia, immunodeficiency and vascular disease were excluded because the prevalence was less than 10%. There was no significant difference on PEEP (9.1±3.7 vs.9.3±3.3 cmH2O; p = 0.71) and FiO2 (0.58±0.19 vs.0.57±0.17; p = 0.69) between survivors and non-survivors (Fig. 1).

**Table 1:** Univariate analysis by mortality.

Variable	Odds ratio	95% CI	P
Gender (male as reference)	0.83	0.46–1.47	0.512
Age	1.04	1.02–1.06	<0.001
BMI	0.99	0.94–1.03	0.521
Cigarette smoking (50.35%)	1.02	0.58–1.81	0.935
Alcohol (56.38%)	0.89	0.50–1.57	0.684
Location (MICU as reference)			
SICU	0.96	0.46–2.00	0.910
Mixed ICU	0.60	0.27–1.33	0.210
Admission type			
Unscheduled surgery	Reference		
Scheduled surgery	1.54	0.38–6.21	0.543
Medical	1.87	0.74–4.69	0.181
Causes of ARDS			
Sepsis	1.44	0.81–2.58	0.217
Transfusion	0.59	0.13–2.71	0.498
Aspiration	1.19	0.63–2.26	0.585
Pneumonia	0.94	0.53–1.67	0.839
Other lung condition	0.31	0.07–1.34	0.117
Admission source			
OR	Reference		
ED	1.95	0.54–7.04	0.305
Floor ward	3.70	1.02–13.46	0.047
Others	2.08	0.53–8.18	0.293
Comorbidity			
Chronic dialysis (3.19%)	1.04	0.21–5.12	0.965
Leukemia (2.13%)	7.68	1.37–43.01	0.020
Immunodeficiency (6.74%)	2.88	1.10–7.52	0.031
Cirrhosis (4.27%)	1.86	0.54–6.40	0.325
Diabetes (21.63%)	1.10	0.56–2.17	0.777
Hypertension (39.15%)	1.20	0.68–2.14	0.530
Myocardial infarction (4.98%)	1.47	0.45–4.87	0.525
Heart failure (3.90%)	2.15	0.61–7.58	0.236
Vascular disease (1.42%)	11.38	1.16–111.42	0.037
Dementia (2.84%)	2.23	0.52–9.63	0.281
Chronic pulmonary disease (5.32%)	1.88	0.62–5.73	0.265
Arthritis (5.69%)	1.22	0.38–3.91	0.743
Peptic ulcer (3.91%)	3.18	0.94–10.82	0.063
Vasopressor use (50%)	5.08	2.60–9.92	<0.001
Hemoglobin (with 1 unit increase)	0.86	0.74–1.01	0.068
WBC	1.00	0.99–1.01	0.375
Platelet count	0.996	0.993–0.999	0.010
Creatinine	1.09	0.93–1.28	0.297
Bilirubin	1.35	1.16–1.57	<0.001
Sodium	1.00	0.95–1.05	0.997
Potassium	1.98	1.27–3.08	0.003
Glucose	1.00	0.99–1.003	0.402
Bicarbonate	0.93	0.88–0.98	0.006
Phosphate	1.40	1.09–1.81	0.009
Magnesium	1.34	0.46–3.88	0.593
Total protein	0.80	0.52–1.24	0.326
Albumin	0.73	0.37–1.46	0.375
Minimal glucose	0.997	0.98–1.01	0.694
FiO2	1.13	0.33–3.81	0.845
PaO2	1.003	0.998–1.008	0.242
PaCO2	0.99	0.97–1.02	0.622
pH	0.028	0.002–0.41	0.009
Lowest temp.	0.70	0.53–0.93	0.014
Highest temp.	0.63	0.45–0.88	0.007
Lowest systolic pressure	0.98	0.96–0.99	0.044
Highest systolic pressure	0.998	0.987–1.009	0.689
Lowest mean blood pressure	0.98	0.95–1.005	0.103
highest mean blood pressure	0.99	0.98–1.001	0.366
Lowest heart rate	0.997	0.982–1.012	0.708
Highest heart rate	1.007	0.994–1.020	0.299
Lowest respiratory rate	1.02	0.98–1.06	0.438
Highest respiratory rate	0.99	0.96–1.02	0.469
Urine output	0.9997	0.9995–0.9999	0.027
Transfusion of RBC (23.05%)	1.55	0.82–2.93	0.178
FFP transfusion	1.43	0.71–2.93	0.316

Abbreviations: OR: operating room; ED, emergency department; WBC, white blood cell; RBC, red blood cell; FFP, fresh frozen plasma.

As a result, a total of 21 covariates were entered into the full model. After stepwise forward selection with p = 0.1, eight covariates remained in the model (Table 2), including age (coefficient: 0.05, 95% CI: 0.027–0.077), vasopressor (coefficient: 1.47, 95% CI: 0.70–2.23), potassium (coefficient: 0.78, 95% CI: 0.22–1.34), platelet (coefficient: -0.005, 95% CI: -0.008, -0.001), hemoglobin (coefficient: -0.20, 95% CI: -0.41–0.004), highest heart rate (coefficient: 0.028, 95% CI: 0.01–0.046), highest temperature (coefficient: -0.45, 95% CI: -0.87, -0.02) and PaO2 (coefficient: 0.007, 95% CI: -0.00021–0.013). These coefficients were obtained by using complete case analysis. After multiple imputations procedure, the estimated coefficients shrunk towards zero for all covariates. The coefficients remained unchanged as compared to the original analysis with bootstrap estimation. At this stage, we would like to use the original complete case analysis as the main effect model because of the parsimony principal in estimation analysis.

**Table 2:** Main effect model after stepwise selection of covariates†.

Covariates	Coefficient (95% confidence interval)			p‡
	Original complete case analysis	Multiple imputation	Shrunken with bootstrap	
Age	0.05 (0.027, 0.077)	0.05 (0.026, 0.075)	0.05 (0.027, 0.077)	<0.001
Vasopressor	1.47 (0.70, 2.23)	1.35 (0.61, 2.09)	1.47 (0.58, 2.36)	<0.001
Potassium	0.78 (0.22, 1.34)	0.69 (0.15, 1.22)	0.78 (0.06, 1.50)	0.012
Platelet	-0.005 (-0.008, -0.001)	-0.004 (-0.008, -0.001)	-0.005 (-0.009, -0.0007)	0.010
Hemoglobin	-0.20 (-0.41, 0.004)	-0.18 (-0.37, 0.022)	-0.20 (-0.41, 0.012)	0.081
Highest heart rate	0.028 (0.01, 0.046)	0.027 (0.009, 0.044)	0.028 (0.008, 0.048)	0.003
Highest temperature	-0.45 (-0.87, -0.02)	-0.43 (-0.85, -0.012)	-0.45 (-0.90, 0.009)	0.044
PaO2	0.007 (-0.00021, 0.013)	0.006 (-0.0004, 0.013)	0.007 (-0.0008, 0.014)	0.064

† Covariates were selected by using stepwise forward selection at a significance level of 0.1.

‡ Coefficients were reported after multiple imputations for missing values.

**Fig 2:**
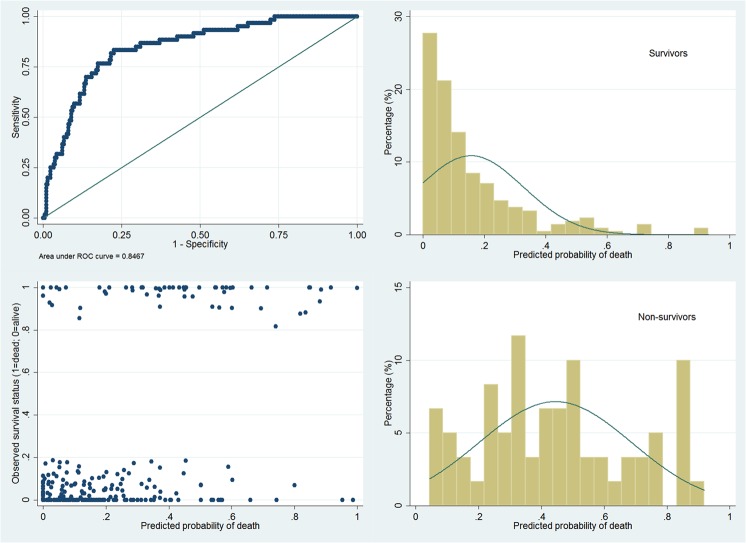
Four diagnostic plots to describe discrimination in a model fit with an area under operating characteristics curve of 0.85. In survivors, the predicted probability of death ($\hat{\pi }$) gathered below 0.2, indicating a good negative predictive power of the model. However, $\hat{\pi }$ scattered more evenly in non-survivors, indicating a limited power of positive predictive value.

**Fig 3:**
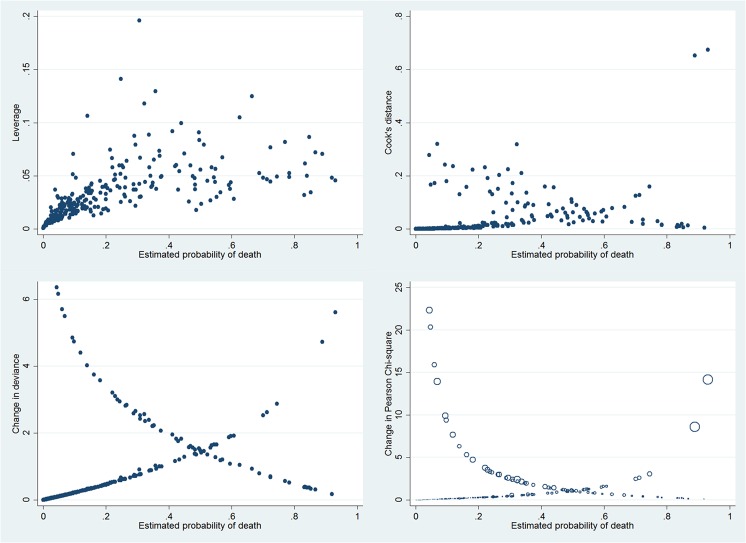
Logistic regression diagnostics plotted against estimated probability of death ($\hat{\pi }$). The upper left shows the leverage versus $\hat{\pi }$. The influence diagnostic Δ$\hat{\beta }$ is plotted versus $\hat{\pi }$ in upper right panel. We noted two points lie somewhat away from the rest of the data. The lower right panel shows the change in Pearson Chi-square as a function of the estimated probability of death, which is helpful in identifying poorest fit points. In this figure, five points are poorly fitted in the top left and top right corner of the plot (Δχ^2^>10). The size of the symbol is proportional to Δ$\hat{\beta }$, allowing us to more clearly ascertain the relative contribution of residual and leverage to Δ$\hat{\beta }$. The largest circle in the right corner correspond to a moderate leverage and a large Δχ^2^, indicating that high leverage might not be a contributing factor. The same five points are shown in lower right panel, but note that the range of Δχ2 is much greater than the change in deviance (ΔD). As a result we identified five covariate patterns with large values of ΔD or Δχ^2^ (poorest fit) and two with outlying values of Δ$\hat{\beta }$ (largest influence).

Interaction terms were evaluated for all possible interactions, which showed no statistically significant interactions among variables. Linearity assumption for continuous variables in the main effect model was assessed by using multiple fractional polynomials, which showed that other non-linear functions were no better than the linear one. As a result we adopted the original main effect model as the final model. Overall model fit was assessed by using Hosmer-Lemeshow goodness-of-fit test, which showed a χ^2^ (df = 8) of 6.54 (p = 0.59). Graphical assessment of the overall fit was shown in Fig. 2. The discrimination of the model was good with an area under operating characteristics curve of 0.85. In survivors, the predicted probability of death ($\hat{\pi }$) gathered below 0.2, indicating a good negative predictive power of the model. However, the $\hat{\pi }$ scattered evenly in non-survivors, indicating a limited power of positive predictive value. Plot of Logistic regression diagnostics versus estimated probability of death ($\hat{\pi }$) were shown in Fig. 3. From the figures we identified five covariate patterns with large values of ΔD or Δχ^2^ (poorest fit) and two with outlying values of Δ$\hat{\beta }$ (largest influence). These covariate patterns (#72, 126, 137, 147, 171, 207) were shown in table 3. Because there were many continuous covariates, one covariate pattern corresponded to one subject. For instance, the subject #137 was characterized by old age, vasopressor use, hyperkalemia and thrombocytopenia, which was a covariate pattern of high probability of death. However, the subject was observed to survive which violated the fitted model and thus it was considered as an outlier. We examined how the exclusion of these outliers could influence the estimation of coefficients (Table 4). The result showed that all coefficients changed significantly after exclusion of outliers. We would like to use this model as the prediction model for probability of death in future ARDS patients requiring mechanical ventilation:

$$\pi \left(Χ\right)\;=\;\frac{e^{g\left(Χ\right)}}{1+e^{g\left(Χ\right)}}$$

where

$$g\left(Χ\right)\;=\;0.06\times age\left(in\;years\right)+2.23\left(if\;on\;vasopressor\right)+1.37\times potassium\;\left(\frac{mmol}{l}\right)-0.007\times platelet\;count\;\left(\times 10^{9}\right)+0.03\times heart\;rate\;\left(/min\right)-0.29\times Hb\left(\frac{g}{dl}\right)-0.67\times T\left(℃\right)+0.01\times PaO_{2}+13.$$

**Table 3:** Covariate patterns that do not fit or have considerable influence on estimated coefficient.

P#†	72	126	137	147	171	207
Mortality (1 = non-survivor)	1	0	0	1	1	1
Age (years)	49	54	89	72	54	43
Vasopressor use	0	1	1	0	0	0
Potassium (mmol/l)	4.70	6.50	6.20	3.90	3.60	3.70
Platelet (×10^9^)	134.00	103.00	40.00	331.00	190.00	103.00
Highest heart rate (/min)	140.00	122.00	72.00	86.00	130.00	140.00
Hemoglobin (g/dl)	14.00	11.20	9.70	10.30	11.30	11.20
Temperature (°C)	38.90	36.40	37.50	37.50	38.50	38.60
PaO2 (mmHg)	68.00	104.00	137.00	75.00	73.00	83.00
$\hat{\pi }$	0.07	0.89	0.93	0.04	0.05	0.06
Δχ^2^	13.93	8.61	14.14	22.31	20.35	15.88
ΔD	5.49	4.73	5.60	6.35	6.16	5.69
leverage	0.02	0.07	0.05	0.01	0.01	0.01
Δ$\hat{\beta }$	0.32	0.65	0.67	0.28	0.17	0.17

Notations: $\hat{\pi }$, estimated probability of death; Δχ^2^, change in Pearson Chi-square; ΔD, change in deviance; Δ$\hat{\beta }$, Cook’s distance.

† The number of covariate pattern is equivalent to the number of observations as there are several continuous covariates. The number itself was meaningless in itself but simply reflected the sequence of enrollment in the dataset.

**Table 4:** Estimated coefficients from all data, the percent change when covariate patterns with poorest fit and largest influence were deleted.

variable	All data coefficients	Percent change from all data coefficient when outlying covariate patterns were deleted		
		Poorest fit 72,137,147,171,207	largest influence 126,137	All six
Age	0.052	0.060 (15.4%)	0.057 (9.6%)	0.061(17.3%)
Vasopressor use	1.469	2.121 (44.4%)	1.600 (8.9%)	2.231(51.9%)
Potassium	0.777	1.071 (37.8%)	1.204 (54.9%)	1.366(75.8%)
Platelet	-0.005	-0.006 (20%)	-0.006 (20%)	-0.007(40%)
Highest heart rate (/min)	0.028	0.029 (3.6%)	0.029 (3.6%)	0.031(10.7%)
Hemoglobin (g/dl)	-0.201	-0.301 (49.8%)	-0.201 (0)	-0.294(46.3%)
Temperature (°C)	-0.446	-0.545 (22.2%)	-0.546 (22.4%)	-0.666(49.3%)
PaO2 (mmHg)	0.007	0.009 (28.6%)	0.008 (14.3%)	0.010(42.9%)
_cons	7.646	10.078 (31.8%)	9.374 (22.6%)	12.965(69.6%)
Model statistics				
Chi-squared	76.92	97.13	88.02	103.61
log likelihood	-105.31	-88.80	-99.26	-85.33
pseudo-R-squared	0.27	0.35	0.31	0.38

In the last column of the table five of the coefficients changed >40%, indicating that deleting all six covariate patterns had a significant effect on estimated coefficients. The six subjects whose patterns of data go against the prediction model should be dropped out from the model, and the final coefficients were as listed in the last column of the table.

**Fig 4:**
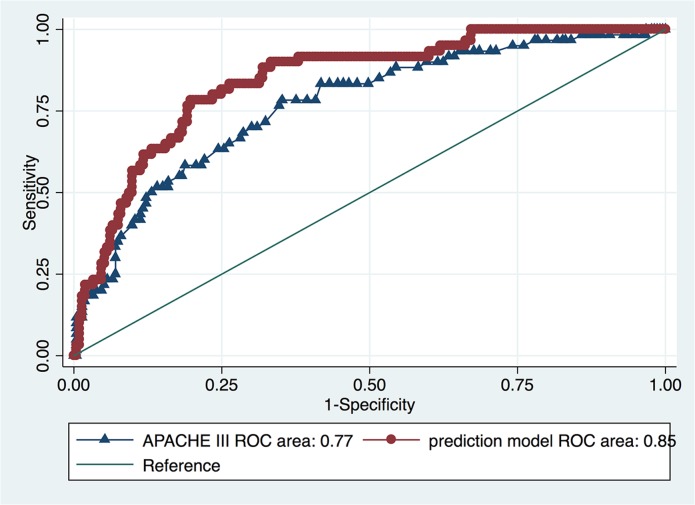
Comparison of area under ROC for APACHE III and prediction model. The result showed that the prediction model had better discrimination than APACHE III (AUC: 0.85, 95% CI: 0.79–0.90 vs AUC: 0.77 95% CI: 0.70–0.84; p = 0.037).

The prediction model was compared with APACHE III score for its discrimination in predicting mortality (Fig. 4). The result showed that the prediction model had better discrimination than APACHE III (AUC: 0.85, 95% CI: 0.79–0.90 vs. AUC: 0.77 95% CI: 0.70–0.84; p = 0.037).

## Discussion

The study, by using a prospectively collected dataset, established a prediction model for ARDS patients requiring mechanical ventilation. The model included eight covariates without interaction and non-linear functions. The parsimony of the model may improve the prediction accuracy for future cohort with similar characteristics.[28] The model discrimination was good as reflected by an area under ROC of 0.85. Parameters incorporated into the model were all collected within 24 hours of ICU entry, allowing for early recognition of patients at high risk of death.

For a prediction model to be clinically useful, it should be easy to use. In the study we incorporated variables that were readily available in routine clinical practice. In the model, we found that old age was a significant independent risk factor for death. This is consistent with other studies and prediction models.[29–31] Vasopressor use was also found to be an independent risk factor for mortality. Vasopressor use indicates circulatory failure which is established to be associated with multiple organ failures (e.g. acute kidney injury) in critically ill patients.[32, 33] Organ failure such as acute kidney injury is a well-known mortality risk factor that is also supported by our previous study.[34] Platelet count was also found to be associated with mortality risk in this cohort. However, our previous study showed that it was platelet distribution width and mean platelet volume, rather than platelet count that were independently associated with mortality risk.[35] In that study, we included unselected critically ill patients, and the mortality was slightly higher. The difference in study population and severity of illness may partly explain the disparity between these two cohorts.

Because the prediction model was established with single cohort without external validation, overfitting is a major concern. We employed bootstraps procedure to shrink coefficient and chose model with the principal of parsimony. However, the result showed that the bootstrap procedure did not change the coefficient, indicating that the estimated coefficient is less likely to be biased. There is no evidence of substantial problem with model fit as reflected by the non-significance of Hosmer-Lemeshow goodness-of-fit test. However, such overall model fit cannot exclude some outlying observations. We therefore further examined model fit over the entire set of covariate patterns. As a result, six covariate patterns showed large values in diagnostic statistics, indicating they are either poorly fitted or influential. After exclusion of these six subjects, the coefficients were substantially changed and we choose to retain the model with the outliers excluded. The strength of this technique is to exclude the influence of minority of outlying covariate patterns. However, the shortcoming is certainly that our model cannot be used for subjects with those covariate patterns.

Several limitations need to be acknowledged in the study. First, the major methodological flaws of this secondary analysis is the use of subjects enrolled in a RCT, instead of using data from an observational cohort of consecutive patients. Patients included in a RCT are a selected population that differs from the common patient with the diagnosis under study. For example, the overall mortality rate of this selected group of patients (21%) is below other figures reported in recent epidemiological studies[4–6]. Second, the present analysis is the lack of a validation cohort to test the model. The trial was stopped early due to futility of the intervention. Thus the sample size was small and the dataset cannot be split to training subset and validation subset. However, we examined the overall model fit, as well as the influence of outliers. Furthermore, the problem of overfitting was addressed by using bootstraps procedure to shrink coefficient and the final model was chosen with the principal of parsimony. Third, the reported model is not specific for ARDS patients. In fact, all predictive variables are not specific for ARDS. Thus it would be interesting to test our prediction model in patients without ARDS. Forth, The study suffers slightly from using a single, local cohort, such that international generalisability is questionable. This can be addressed by validating our prediction model in ARDS cohorts from other institutions.

In aggregate, the present study established a prediction model for ARDS patients requiring mechanical ventilation. The model contained eight covariates that are readily available in routine clinical practice and can be applied to all critical care settings. Interaction terms or non-linear functions are not included in the model for parsimony.
